# Hypnotics and injuries among older adults with Parkinson’s disease: a nested case–control design

**DOI:** 10.1186/s12877-023-03944-9

**Published:** 2023-05-01

**Authors:** Takako Fujita, Akira Babazono, Yunfei Li, Aziz Jamal, Sung-a Kim

**Affiliations:** 1 Department of Health Sciences, Faculty of Medical Sciences, Kyushu University, 3-1-1 Maidashi, Higashi-ku, Fukuoka, 812-8582 Japan; 2 Department of Healthcare Administration and Management, Graduate School of Medical Sciences, Kyushu University, Fukuoka, Japan; 3 Department of Epidemiology and Prevention, Center for Clinical Sciences, National Center for Global Health and Medicine, Tokyo, Japan; 4 Health Administration Program, Department of International Business and Management, Faculty of Business and Management, Universiti Teknologi MARA, Selangor Campus, Shah Alam, Malaysia; 5 St. Mary’s Research Center, Kurume, Japan

**Keywords:** Parkinson’s disease, Hypnotics, Benzodiazepines, Orexin receptor antagonists, Melatonin receptor agonists, Injuries, Fractures

## Abstract

**Background:**

Patients with Parkinson’s disease often experience sleep disorders. Hypnotics increase the risk of adverse events, such as injuries due to falls. In this study, we evaluated the association between hypnotics and injuries among older adults with Parkinson’s disease.

**Methods:**

The study used a nested case–control design. The participants were 5009 patients with Parkinson’s disease aged ≥ 75 years based on claims data between April 2016 and March 2019 without prescription hypnotics 1 year before the study started. Hypnotics prescribed as oral medications included benzodiazepines, non-benzodiazepines, orexin receptor antagonists, and melatonin receptor agonists. The incidences of outcomes, including injuries, fractures, and femoral fractures, were determined. Each case had four matched controls. Conditional logistic regression analyses were performed to calculate the odds ratios and 95% confidence intervals for the number of hypnotics taken per day for each type of hypnotic.

**Results:**

The proportion of participants taking at least one type of hypnotic was 18.6%, with benzodiazepines being the most common. The incidence of injuries, fractures, and femoral fractures was 66.7%, 37.8%, and 10.2%, respectively. Benzodiazepines significantly increased the risk of injuries (odds ratio: 1.12; 95% confidence interval: 1.03–1.22), and melatonin receptor agonists significantly increased the risk of femoral fractures (odds ratio: 2.84; 95% confidence interval: 1.19–6.77).

**Conclusions:**

Benzodiazepines and non-benzodiazepines, which are not recommended according to current guidelines, were the most prevalent among older adults with Parkinson’s disease. Benzodiazepines significantly increased the risk of injuries, and melatonin receptor agonists significantly increased the risk of femoral fractures.

**Supplementary Information:**

The online version contains supplementary material available at 10.1186/s12877-023-03944-9.

## Background

In 2016, the number of patients with Parkinson’s disease (PD) worldwide was approximately 6.1 million, and the prevalence increases with age [1]. Patients with PD experience not only motor symptoms, such as tremors and rigid muscles, but also non-motor symptoms, such as sleep disorders, including insomnia, daytime somnolence, and sleep-related movement disorders. These symptoms appear during the early stage of PD, and their frequency increases with disease progression [2–4]. Clinical practice guidelines for PD in Japan suggest pharmacotherapy, phototherapy, and cognitive behavioral therapy for the treatment of sleep disorders in patients with PD. However, there is currently insufficient evidence on the efficacy of any therapy [5].

In a previous cross-sectional study in Sweden, patients using antiparkinsonian agents had a significantly higher fall risk (odds ratio [OR]: 1.68) than patients who did not use antiparkinsonian agents when they used hypnotics [6]. Although the results suggested that using both antiparkinsonian agents and hypnotics might increase the risk of falls, the effect of hypnotics among patients with PD was not clear. Hypnotics are classified into barbiturates, benzodiazepines, non-benzodiazepines, melatonin receptor agonists, and orexin receptor antagonists. According to the 2019 Beers Criteria published by the American Geriatrics Society, barbiturates, benzodiazepines, and non-benzodiazepines should not be administered to older adults [7]. Similarly, the Guidelines for Medical Treatment and Its Safety in the Elderly 2015 published by the Japan Geriatrics Society suggest that benzodiazepines should not be administered to older adults because of potential side effects, whereas non-benzodiazepines may be administered with care in small doses, but they should not be used long-term. Guidelines also report that barbiturates have not been used in recent years [8]. Medical fees are deducted from the standard fee by the Japanese government when multiple psychotropic drugs, including hypnotics, are prescribed or when benzodiazepines are prescribed for more than 1 year. Although there is insufficient evidence for the effectiveness of eszopiclone for the treatment of insomnia in patients with PD, the Movement Disorder Society suggested that eszopiclone can improve global and sleep outcomes associated with insomnia. However, eszopiclone is associated with infrequent but serious injuries, such as fractures [9]. Therefore, the preference to prescribe this medication depends on the physician. Effectively treating sleep disorders is crucial for improving the quality of life of patients with PD and their caregivers.

Older adults have multimorbidity [10]. In Japan, the proportion of adults aged ≥ 75 years with more than two diseases, including non-communicable diseases, is 80.2%, while 64.6% have more than three diseases [11]. Moreover, another report revealed that most patients with PD have several comorbidities [12]. Therefore, older adults with PD may have multimorbidity, which may include diseases that put these patients at a high risk of falls and fractures.

To date, the risk of adverse events, such as injuries, after prescribing hypnotics has not been evaluated among patients with PD. Additionally, medical history information that should be considered when prescribing hypnotics to patients with PD is currently uncertain. Therefore, we evaluated the effects of hypnotics and medical history on injuries among older adults with PD.

## Methods

### Data

We used healthcare claims data from the Latter-Stage Elderly Healthcare Insurance (LSEHI) and long-term care claims data from the Long-Term Care Insurance (LTCI) in Fukuoka Prefecture, Japan. In Japan, all citizens are covered by healthcare insurance systems. Citizens aged ≥ 75 years are enrolled in the LSEHI across the 47 residential areas of Japan. The LTCI is public insurance applicable to all citizens aged ≥ 40 years who require long-term care.

### Study participants

Patients with PD were defined as those who had been diagnosed with PD and were taking antiparkinsonian agents according to healthcare claims data in the 2015 fiscal year (from April 1, 2015, to March 31, 2016). We included patients aged ≥ 75 years as of April 1, 2016, and excluded participants who stayed in medical institutes for ≥ 28 days or long-term care facilities in March 2016.

### Study design and statistical analyses

To determine the prevalence of hypnotic prescriptions, we extracted prescription data by type of hypnotic between April 2016 and March 2019. The hypnotics assessed in this study included the following oral drugs that had been approved in Japan by 2018: benzodiazepines (brotizolam, etizolam, flunitrazepam, triazolam, rilmazafone, nitrazepam, estazolam, quazepam, lormetazepam, haloxazolam, and flurazepam), non-benzodiazepines (zolpidem, eszopiclone, and zopiclone), melatonin receptor agonists (ramelteon), and orexin receptor antagonists (suvorexant). Barbiturate and non-barbiturate hypnotics were not evaluated because they are rarely prescribed in Japan.

We used a nested case–control design, with injuries as the outcome measure. The risk of injuries after being prescribed hypnotics was evaluated from April 2016 to March 2019 among participants who had not been prescribed hypnotics between April 2015 and March 2016. Because injuries included fractures, we evaluated all fractures as well as all types of femoral fracture specifically, because previous studies have shown that patients with PD are at a high risk of fractures, especially hip fractures [13, 14]. The follow-up of patients began from the first prescription of each type of hypnotic. The censor was the loss of qualification for the LSEHI in Fukuoka Prefecture because of death or moving to other prefectures. We extracted the number of prescribed hypnotics by type during the follow-up period and calculated the number of hypnotics per day. The claims data did not report the actual daily dose taken by patients; moreover, some patients only took medications as needed. The variables included sex, age (categorized in 5-year increments), long-term care level, resident facility (own home or retirement home), years after PD diagnosis (less than 1 year, 1 to < 5 years, 5 to < 10 years, and ≥ 10 years), and comorbidities (including injuries, cancer, ischemic heart disease, cerebrovascular disease, dyslipidemia, diabetes mellitus, dementia, osteoporosis, and anemia). The comorbidities were extracted for the year before the beginning of the study (from April 2015 to March 2016) using the codes of the International Classification of Diseases, Tenth Revision, which are provided in the Supplementary Materials 1. The long-term care level was categorized into seven levels: none, requiring some care, and long-term care (divided into five levels, with a higher level indicating more care). Each case was matched to four controls who had not experienced any outcomes of interest by sex, age, long-term care level, residential facility, years after PD diagnosis, and number of days of follow-up using risk-set sampling. Conditional logistic regression analyses were performed to calculate the ORs and 95% confidence intervals (CIs) for the number of hypnotics prescribed per day for each type of hypnotic and comorbidity.

Microsoft SQL Server Management Studio 18 (Microsoft, Washington, US) was used to extract the data, and Stata BE 17.0 (StataCorp LLC, College Station, TX, US) was used for the analyses.

The study was approved by the Institutional Review Board of Kyushu University (Clinical Bioethics Committee of the Graduate School of Healthcare Sciences, Kyushu University).

### Data Availability

The data that support the findings of this study are available from the LSEHI and LTCI in Fukuoka. However, restrictions apply to the availability of these data, which were used under license for the current study. Therefore, the data are not publicly available. Nevertheless, the data are available from the authors upon reasonable request and with permission from these insurance companies.

## Results

**Fig. 1:**
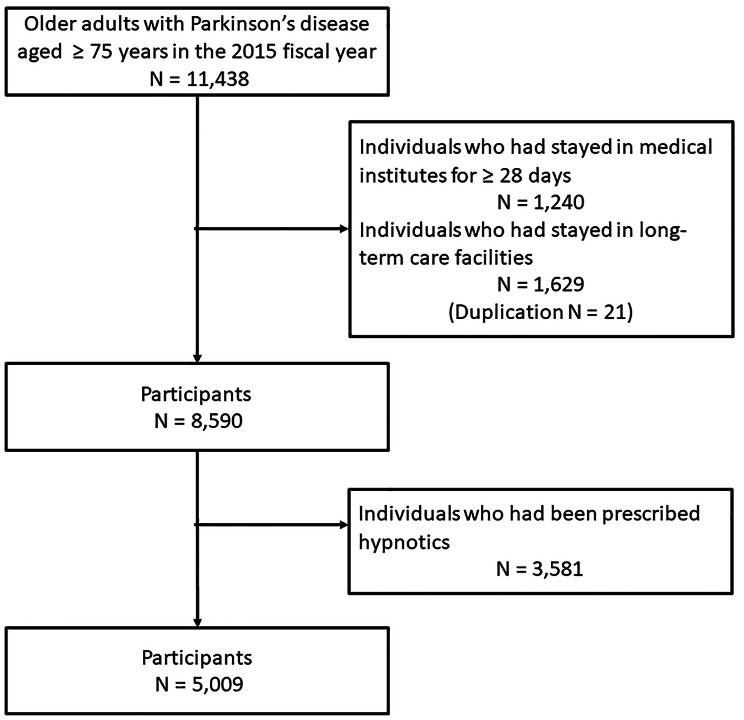
Inclusion criteria and number of patients The total number of patients with PD which excluded individuals who had stayed in medical institutes or long-term care facilities before the study commencement was 8590. The final number of participants, which excluded those who had been prescribed hypnotics during the year before the study commencement, was 5009

The total number of patients with PD was 8590, which included 3581 patients (41.7%) who had been prescribed hypnotics during the year before study commencement. The final number of participants, which excluded those who had been prescribed hypnotics during the year before study commencement, was 5009 (Fig. 1). The proportion of patients who had been prescribed at least one type of hypnotic was 18.6%. Of the various hypnotics prescribed, benzodiazepines were the most common (8.2%), followed by non-benzodiazepines (8.1%).

The proportion of participants with injuries, fractures, and femoral fractures was 66.7%, 37.8%, and 10.2%, respectively. The results of each variable by outcome are shown in Table 1.

**Table 1:** Participant demographics and outcomes

	Injuries					Fractures					Femoral fractures					
	Yes	(%)	No	(%)	p	Yes	(%)	No	(%)	p	Yes	(%)	No	(%)	p	Total
Total	3343	(66.7)	1666	(33.3)		1894	(37.8)	3115	(62.2)		512	(10.2)	4497	(89.8)		5009
Sex					0.000					0.000					0.000	
Male	1324	(62.9)	782	(37.1)		584	(27.7)	1522	(72.3)		121	(5.7)	1985	(94.3)		2106
Female	2019	(69.5)	884	(30.5)		1310	(45.1)	1593	(54.9)		391	(13.5)	2512	(86.5)		2903
Age (years)					0.206					0.754					0.000	
75–79	1138	(66.9)	563	(33.1)		637	(37.4)	1064	(62.6)		136	(8.0)	1565	(92.0)		1701
80–84	1180	(66.7)	589	(33.3)		670	(37.9)	1099	(62.1)		163	(9.2)	1606	(90.8)		1769
85–89	736	(68.3)	342	(31.7)		420	(39.0)	658	(61.0)		151	(14.0)	927	(86.0)		1078
≥ 90	289	(62.7)	172	(37.3)		167	(36.2)	294	(63.8)		62	(13.4)	399	(86.6)		461
Years after Parkinson’s disease diagnosis					0.030					0.081					0.002	
< 1	205	(73.7)	73	(26.3)		115	(41.4)	163	(58.6)		31	(11.2)	247	(88.8)		278
1 to < 5	2123	(65.7)	1108	(34.3)		1185	(36.7)	2046	(63.3)		311	(9.6)	2920	(90.4)		3231
5 to < 10	790	(68.2)	368	(31.8)		468	(40.4)	690	(59.6)		148	(12.8)	1010	(87.2)		1158
≥ 10	225	(65.8)	117	(34.2)		126	(36.8)	216	(63.2)		22	(6.4)	320	(93.6)		342
Long-term care level					0.000					0.000					0.000	
None	1179	(64.7)	643	(35.3)		627	(34.4)	1195	(65.6)		112	(6.1)	1710	(93.9)		1822
Some help required	442	(75.0)	147	(25.0)		292	(49.6)	297	(50.4)		76	(12.9)	513	(87.1)		589
Care level 1	455	(70.7)	189	(29.3)		262	(40.7)	382	(59.3)		77	(12.0)	567	(88.0)		644
Care level 2	488	(74.2)	170	(25.8)		279	(42.4)	379	(57.6)		95	(14.4)	563	(85.6)		658
Care level 3	348	(68.6)	159	(31.4)		205	(40.4)	302	(59.6)		64	(12.6)	443	(87.4)		507
Care level 4	264	(59.6)	179	(40.4)		145	(32.7)	298	(67.3)		48	(10.8)	395	(89.2)		443
Care level 5	167	(48.3)	179	(51.7)		84	(24.3)	262	(75.7)		40	(11.6)	306	(88.4)		346
Place of residence					0.052					0.606					0.000	
Home	3030	(67.2)	1481	(32.8)		1711	(37.9)	2800	(62.1)		429	(9.5)	4082	(90.5)		4511
Retirement home	313	(62.9)	185	(37.1)		183	(36.7)	315	(63.3)		83	(16.7)	415	(83.3)		498
Medical history																
Injury	1784	(82.4)	382	(17.6)	0.000	1157	(53.4)	1009	(46.6)	0.000	329	(15.2)	1837	(84.8)	0.000	2166
Fracture	956	(88.7)	122	(11.3)	0.000	816	(75.7)	262	(24.3)	0.000	246	(22.8)	832	(77.2)	0.000	1078
Femoral fracture	202	(89.4)	24	(10.6)	0.000	181	(80.1)	45	(19.9)	0.000	161	(71.2)	65	(28.8)	0.000	226
Cancer	424	(66.8)	211	(33.2)	0.985	239	(37.6)	396	(62.4)	0.923	56	(8.8)	579	(91.2)	0.212	635
Ischemic heart disease	973	(68.0)	457	(32.0)	0.216	530	(37.1)	900	(62.9)	0.490	149	(10.4)	1281	(89.6)	0.770	1430
Cerebrovascular disease	1569	(65.5)	828	(34.5)	0.065	858	(35.8)	1539	(64.2)	0.005	227	(9.5)	2170	(90.5)	0.093	2397
Dyslipidemia	1469	(67.9)	696	(32.1)	0.145	828	(38.2)	1337	(61.8)	0.581	197	(9.1)	1968	(90.9)	0.022	2165
Diabetes mellitus	1080	(66.7)	539	(33.3)	0.973	571	(35.3)	1048	(64.7)	0.010	146	(9.0)	1473	(91.0)	0.052	1619
Dementia	1167	(64.4)	644	(35.6)	0.009	646	(35.7)	1165	(64.3)	0.019	228	(12.6)	1583	(87.4)	0.000	1811
Osteoporosis	1467	(75.2)	483	(24.8)	0.000	995	(51.0)	955	(49.0)	0.000	271	(13.9)	1679	(86.1)	0.000	1950
Anemia	743	(69.1)	332	(30.9)	0.062	424	(39.4)	651	(60.6)	0.214	127	(11.8)	948	(88.2)	0.052	1075

The results of each type of hypnotic prescribed before the incidence of outcomes are shown in Table 2, where a prescription was defined as more than a one-time prescription. The results show that the proportion of participants with each outcome who had been prescribed hypnotics was lower than those who had not been prescribed any hypnotics.

**Table 2:** Proportion of patients with each type of hypnotic prescribed before the incidence of each outcome

		Injury					
		Yes	(%)	No	(%)	*P*	Total
		3343	(66.7)	1666	(33.3)		5009
Benzodiazepines						0.000	
	Yes	110	(53.4)	96	(46.6)		206
	No	3233	(67.3)	1570	(32.7)		4803
Non-benzodiazepines						0.000	
	Yes	99	(47.8)	108	(52.2)		207
	No	3244	(67.6)	1558	(32.4)		4802
Melatonin receptor agonists						0.009	
	Yes	43	(53.1)	38	(46.9)		81
	No	3300	(67.0)	1628	(33.0)		4928
Orexin receptor antagonists						0.000	
	Yes	43	(43.4)	56	(56.6)		99
	No	3300	(67.2)	1610	(32.8)		4910
		**Fracture**					
		**Yes**	**(%)**	**No**	**(%)**	***P***	**Total**
		1894	(37.8)	3115	(62.2)		5009
Benzodiazepines						0.000	
	Yes	67	(23.3)	221	(76.7)		288
	No	1827	(38.7)	2894	(61.3)		4721
Non-benzodiazepines						0.000	
	Yes	67	(24.3)	209	(75.7)		276
	No	1827	(38.6)	2906	(61.4)		4733
Melatonin receptor agonists						0.000	
	Yes	29	(22.0)	103	(78.0)		132
	No	1865	(38.2)	3012	(61.8)		4877
Orexin receptor antagonists						0.000	
	Yes	30	(19.4)	125	(80.6)		155
	No	1864	(38.4)	2990	(61.6)		4854
		**Femoral fracture**					
		**Yes**	**(%)**	**No**	**(%)**	***P***	**Total**
		512	(10.2)	4497	(89.8)		5009
Benzodiazepines						0.007	
	Yes	24	(6.2)	362	(93.8)		386
	No	488	(10.6)	4135	(89.4)		4623
Non-benzodiazepines						0.008	
	Yes	23	(6.2)	347	(93.8)		370
	No	489	(10.5)	4150	(89.5)		4639
Melatonin receptor agonists						0.109	
	Yes	12	(6.7)	168	(93.3)		180
	No	500	(10.4)	4329	(89.6)		4829
Orexin receptor antagonists						0.009	
	Yes	10	(4.8)	197	(95.2)		207
	No	502	(10.5)	4300	(89.5)		4802

After matching using risk-set sampling, all of the cases were matched to the four controls. The results of the conditional logistic regression analyses performed for each outcome and the number of hypnotics prescribed per day by hypnotic type are shown in Table 3. Benzodiazepines significantly increased the risk of injuries (OR: 1.12; 95% CI: 1.03–1.22). Melatonin receptor agonists significantly increased the risk of femoral fractures (OR: 2.84; 95% CI: 1.19–6.77). Having a history of injuries was more strongly associated with each outcome for all types of hypnotic than having no history of injuries. Having a history of osteoporosis significantly increased the incidence of injuries and fractures, and having a history of anemia significantly increased the incidence of injuries, except in those prescribed benzodiazepines. Moreover, having a history of cancer increased the incidence of injuries in those prescribed orexin receptor antagonists, while having a history of cerebrovascular disease significantly lowered the risk of fractures in those prescribed non-benzodiazepines and melatonin receptor agonists. Other medical histories did not show significant differences.

**Table 3:** Results of the conditional logistic regression analysis for each type of hypnotic and each outcome

	Injuries		Fractures		Femoral fractures	
	OR (95% CI)		OR (95% CI)		OR (95% CI)	
**Benzodiazepines**						
Number of hypnotics per day	1.12	(1.03–1.22)	1.17	(0.89–1.56)	1.99	(0.95–4.18)
Injury/fracture/femoral fracture	2.93	(2.68–3.19)	5.50	(4.81–6.29)	16.75	(11.39–24.62)
Cancer	1.11	(0.98–1.25)	1.13	(0.95–1.35)	0.91	(0.62–1.33)
Ischemic heart disease	1.05	(0.96–1.15)	0.98	(0.87–1.12)	1.12	(0.86–1.44)
Cerebrovascular disease	0.93	(0.86–1.01)	0.91	(0.81–1.02)	0.83	(0.66–1.05)
Dyslipidemia	0.98	(0.90–1.06)	0.95	(0.84–1.06)	0.82	(0.65–1.04)
Diabetes mellitus	0.99	(0.91–1.09)	0.91	(0.80–1.03)	1.02	(0.79–1.31)
Dementia	0.99	(0.90–1.09)	0.95	(0.84–1.08)	1.08	(0.84–1.40)
Osteoporosis	1.32	(1.21–1.45)	1.27	(1.12–1.44)	1.09	(0.86–1.39)
Anemia	1.07	(0.97–1.19)	0.98	(0.86–1.13)	0.87	(0.66–1.15)
**Non-benzodiazepines**						
Number of hypnotics per day	1.21	(0.97–1.51)	1.25	(0.99–1.59)	1.16	(0.49–2.71)
Injury/fracture/femoral fracture	2.95	(2.71–3.22)	5.85	(5.09–6.73)	17.96	(12.17–26.51)
Cancer	1.10	(0.97–1.24)	1.17	(0.98–1.39)	0.99	(0.67–1.44)
Ischemic heart disease	1.03	(0.94–1.12)	1.03	(0.91–1.17)	1.18	(0.91–1.53)
Cerebrovascular disease	0.95	(0.87–1.03)	0.86	(0.77–0.96)	0.81	(0.64–1.02)
Dyslipidemia	0.95	(0.88–1.04)	0.92	(0.82–1.03)	0.92	(0.72–1.16)
Diabetes mellitus	0.97	(0.89–1.06)	0.97	(0.85–1.09)	1.00	(0.78–1.29)
Dementia	1.03	(0.94–1.13)	0.99	(0.87–1.12)	1.23	(0.95–1.58)
Osteoporosis	1.35	(1.23–1.48)	1.27	(1.13–1.44)	1.23	(0.96–1.57)
Anemia	1.12	(1.01–1.23)	0.91	(0.79–1.05)	0.82	(0.62–1.08)
**Melatonin- receptor agonists**						
Number of hypnotics per day	1.03	(0.99–1.08)	1.15	(1.00–1.31)	2.84	(1.19–6.77)
Injury/fracture/femoral fracture	2.92	(2.68–3.19)	5.86	(5.10–6.74)	19.67	(13.18–29.35)
Cancer	1.11	(0.98–1.25)	1.15	(0.97–1.37)	0.71	(0.48–1.04)
Ischemic heart disease	1.05	(0.95–1.14)	1.05	(0.92–1.19)	1.09	(0.84–1.40)
Cerebrovascular disease	0.94	(0.87–1.03)	0.86	(0.77–0.97)	0.83	(0.66–1.05)
Dyslipidemia	0.94	(0.87–1.03)	0.96	(0.85–1.08)	0.90	(0.71–1.15)
Diabetes mellitus	0.98	(0.90–1.07)	0.95	(0.84–1.08)	1.02	(0.79–1.31)
Dementia	0.99	(0.90–1.08)	0.96	(0.84–1.09)	1.19	(0.92–1.54)
Osteoporosis	1.32	(1.20–1.44)	1.23	(1.09–1.40)	1.23	(0.96–1.57)
Anemia	1.11	(1.00–1.22)	0.92	(0.80–1.05)	0.84	(0.64–1.12)
**Orexin- receptor antagonists**						
Number of hypnotics per day	1.43	(0.97–2.11)	1.13	(0.82–1.55)	1.19	(0.32–4.51)
Injury/fracture/femoral fracture	2.98	(2.73–3.25)	5.79	(5.06–6.63)	15.62	(10.75–22.69)
Cancer	1.15	(1.02–1.31)	1.14	(0.96–1.35)	0.93	(0.64–1.35)
Ischemic heart disease	1.06	(0.97–1.16)	0.98	(0.86–1.11)	0.98	(0.76–1.26)
Cerebrovascular disease	0.93	(0.85–1.01)	0.95	(0.85–1.07)	0.98	(0.77–1.23)
Dyslipidemia	0.97	(0.89–1.06)	0.92	(0.82–1.03)	0.83	(0.66–1.06)
Diabetes mellitus	0.96	(0.88–1.05)	0.94	(0.83–1.07)	0.98	(0.76–1.26)
Dementia	0.98	(0.89–1.08)	1.01	(0.88–1.15)	1.08	(0.84–1.38)
Osteoporosis	1.30	(1.19–1.43)	1.24	(1.10–1.41)	1.14	(0.89–1.45)
Anemia	1.11	(1.01–1.23)	0.99	(0.86–1.13)	0.96	(0.73–1.26)

OR, odds ratio; CI, confidence interval. Each disease variable was defined as the medical history before the beginning of the study

## Discussion

We evaluated the relationship between hypnotics and injuries in older adults with PD. Approximately half of the older patients with PD had been prescribed hypnotics. Among the participants, benzodiazepines and non-benzodiazepines were the most prevalent, which are not recommended according to current guidelines [7, 8]. Additionally, we showed that benzodiazepines significantly increased the risk of injuries, and melatonin receptor agonists significantly increased the risk of femoral fractures.

Current guidelines [7–9] do not discourage the use of melatonin receptor agonists, which seem to be safer than other hypnotics. Although some countries offer melatonin as an over-the-counter drugs or supplements, it is not available in Japan. Previous studies have shown that ramelteon, a melatonin receptor agonist, does not have a significant effect on falls or fractures [15–17]. However, we found that melatonin receptor agonists increased the risk of femoral fractures in patients with PD, which may be attributed to physician bias, where melatonin receptor agonists are prescribed to patients who are at a high risk of falls because these drugs are less likely than benzodiazepines and non-benzodiazepines to cause falls. However, the OR was 2.84, which is considered high, even when indication bias is considered. Because melatonin receptor agonists can be used in patients with PD [18], and ramelteon has been shown to be effective for sleep disturbances in patients with PD [19], these drugs may be preferred for the treatment of sleep disorders. Patients with PD are at a higher risk of fractures, especially hip fractures, than patients without PD [13, 14]. This might have affected the results showing that melatonin receptor agonists significantly increase the risk of femoral fractures among patients with PD. Our findings suggest that physicians should carefully assess the condition of patients and that melatonin receptor agonists should be avoided in patients who are at a high risk of experiencing adverse events due to hypnotics. However, the number of participants prescribed melatonin receptor agonists was lower than those prescribed other types of medication. Only one type of melatonin receptor agonist was approved in Japan during the study period; therefore, future research is required.

Orexin receptor antagonists are considered to be as safe as melatonin receptor agonists. A previous study in older adults showed that suvorexant, an orexin receptor antagonist, is associated with a lower risk of falls than placebo; however, somnolence was more common with suvorexant than with placebo [20]. Another previous study demonstrated that lemborexant, another orexin receptor antagonist that was approved in Japan in 2020, significantly lowered the risk of falls [17]. In contrast, we found that orexin receptor antagonists did not increase the risk of injuries. However, few studies have evaluated the adverse events of orexin receptor antagonists; therefore, further studies are required.

A large proportion of patients in our study were prescribed benzodiazepines, which significantly increased the risk of injuries. However, current guidelines advise that benzodiazepines should be avoided in older adults. Moreover, medical fees in Japan are deducted from the standard when multiple psychotropic drugs, including hypnotics, are prescribed or when benzodiazepines are prescribed for more than 1 year. Chronic benzodiazepine use is associated with a significantly greater risk of fractures than intermittent benzodiazepine use [21]. We observed a similar trend, where the risk of injuries increased with the increase in the number of benzodiazepines prescribed per day, although the risk of fractures was not significantly different. Therefore, patients who are at a low risk of experiencing adverse events induced by hypnotics may be prescribed benzodiazepines. Taken together, although the risk of milder injuries may be significantly higher with benzodiazepine use, the risk of fractures, which considerably impact activities of daily living, was not significantly increased by benzodiazepines. To adhere to guidelines and minimize adverse events, including injuries, it is crucial to determine the reasons for prescribing benzodiazepines. Although similar to benzodiazepines, non-benzodiazepines are discouraged in older adults because of the risk of falls and fractures; however, we did not observe significant differences in outcomes. Eszopiclone for the treatment of insomnia in patients with PD showed a similar safety profile to that of placebo, although the number of patients prescribed this drug was only 15 [22]. We showed a similar result; therefore, we suggest that non-benzodiazepines may be prescribed following an assessment of the risk of adverse events by a physician and informing patients and their families of these risks. Previous studies have found that hypnotics increase the risk of pneumonia, except for pneumonia caused by viruses, as well as the risk of cognitive and physical impairment due to trauma and pressure ulcers [23, 24]. Therefore, assessing the risk of such adverse events is crucial when prescribing hypnotics.

We also evaluated the associations of injuries and medical history with hypnotics in older adults with PD. We found that histories of injuries, osteoporosis, and anemia significantly increased the risk of injuries. Patients with PD are at a higher risk of fractures than those without PD, and this risk increases if they have previously experienced fractures [14], which is in line with our results. Fractures are associated with osteoporosis, and patients with PD are at a significantly higher risk of developing osteoporosis [25]. Furthermore, osteoporosis is caused by non-communicable diseases, such as ischemic heart disease, dyslipidemia, and diabetes mellitus [26–28], and patients with diabetes mellitus [29] or cancer [30] have a higher risk of falls. We found that osteoporosis significantly increased the risk of fractures, although non-communicable diseases did not significantly influence the risk of injuries. In addition, having a history of cancer or anemia significantly increased the risk of injuries in those prescribed orexin receptor antagonists. A previous study reported that patients with dementia are more likely to experience fractures following falls after using suvorexant [31]. However, we did not observe significant differences between participants with and without dementia, which may be because there were few patients who had been prescribed orexin receptor antagonists and had experienced injuries. A history of cerebrovascular disease reduced the risk of fractures in those prescribed non-benzodiazepines and melatonin receptor agonists, but not in those prescribed other hypnotics. Other outcomes did not show significant differences in risk with ORs of < 1. There might be selection biases, meaning that these medications were more likely to be prescribed in severely disabled stroke patients, who cannot walk anymore and therefore are at a reduced risk of fractures. A previous study reported that the proportion of stroke patients with fractures increases over time [32], which is inconsistent with our results. Patients with PD with a history of cerebrovascular disease are at a high risk of experiencing falls. Therefore, physicians should prescribe hypnotics only to patients who are independent and who have mild cerebrovascular disease to ensure that injuries do not increase following the use of hypnotics. Thus, it is crucial that the medical history of patients is considered and that hypnotics are not prescribed to patients who have experienced osteoporosis or injuries. Additionally, it would be valuable for both physicians and patients if the guidelines for prescribing hypnotics to patients with PD would highlight the importance of medical history.

Because PD is a progressive disease, the risk of falls increases over time. Even in patients who do not experience adverse events due to hypnotics, risk assessments should be conducted as needed, and safer treatments should be considered. Cognitive behavioral therapy is a treatment option for patients with sleep disorders, and small-scale studies have suggested that it is effective in improving sleep in patients with PD with sleep disorders [33–35]. In patients with PD who are at a high risk of falls, cognitive behavioral therapy may be more appropriate than pharmacotherapy for improving their quality of life.

This study has several limitations. First, information on the dose and frequency of hypnotic use were unavailable from the claims data. Therefore, in this study, we analyzed the number of hypnotics prescribed per day. Moreover, the insurance data did not include information on PD severity; therefore, the long-term care level was assessed as an alternative. Second, we did not include variables that may influence hypnotic-induced adverse events, such as family members, body mass index, and nutrition status. However, we did randomly match patients with control subjects, which would have minimized this bias. Finally, the risk of falls before hypnotics were prescribed was not evaluated, and melatonin receptor agonists and orexin receptor antagonists may have been prescribed to participants at a high risk of falls. Therefore, a larger-scale study in a clinical setting to evaluate the risk of falls before hypnotics are prescribed is necessary.

## Conclusions

Approximately half of the older patients with PD had been prescribed hypnotics in this study. Among the study participants, benzodiazepines and non-benzodiazepines were the most prevalent, which are not recommended according to current guidelines. Additionally, benzodiazepines significantly increased the risk of injuries, and melatonin receptor agonists significantly increased the risk of femoral fractures. For safety reasons, physicians may prescribe melatonin receptor agonists to patients who are at a high risk of adverse events. However, our findings suggest that a comprehensive assessment of older patients with Parkinson’s disease is crucial before prescribing any type of hypnotic.

## Electronic supplementary material

Below is the link to the electronic supplementary material.

Supplementary Material 1

## Data Availability

The data that support the findings of this study are available from the Fukuoka Prefecture Wide-Area Association of the Latter-Stage Elderly Healthcare Insurance and the Fukuoka Prefecture Wide-Area Association of the Long-term Care Insurance, but restrictions apply to the availability of these data, which were used under license for the current study and so are not publicly available. Data are, however, available from the authors on reasonable request and with permission of these insurance companies.

## Abbreviations

PD: Parkinson’s disease
LSEHI: Latter-Stage Elderly Healthcare Insurance
LTCI: Long-Term Care Insurance
OR: Odds ratio
CI: Confidence interval
