# Supplementary material for: Hypnotics and injuries among older adults with Parkinson’s disease: a nested case–control design
Source: BMC Geriatr. 2023 May 1;23:259. doi: 10.1186/s12877-023-03944-9 (PMC10152606; doi:10.1186/s12877-023-03944-9)
Supplement: Supplementary file 1 — Supplementary Material 1 [file 12877_2023_3944_MOESM1_ESM.docx]

**Supplemental File**

International Classification of Diseases, Tenth Revision, codes and individual diagnosis codes to extract comorbidities

| Disease | ICD-10 code | Individual diagnosis code |
| --- | --- | --- |
| Injury | S, T0–T14 |  |
| Fracture | S02, S12, S22, S32, S42, S52, S62, S72, S82, S92, T02, T08, T10, T12 |  |
| Femoral fracture | S72 |  |
| Cancer | C |  |
| Ischemic heart disease | I20–I25 |  |
| Cerebrovascular disease | I6 |  |
| Dyslipidemia | E78 |  |
| Diabetes mellitus | E10–E14 |  |
| Dementia | G30, F010, F011 (excepting CADASIL and CARASIL), F012, F019, F03 (only with dementia) | 8845840: Dementia with Lewy Bodies  8850077: Parkinson’s disease with dementia  8844891: Frontotemporal dementia  8848534: Argyrophilic Granular Dementia |
| Osteoporosis | M80–M82 |  |
| Anemia | D5–D64 |  |

ICD, International Classification of Diseases
